# Catheter, surgical, or hybrid procedure: what future for atrial fibrillation ablation?

**DOI:** 10.1186/s13019-021-01565-0

**Published:** 2021-06-26

**Authors:** Giuseppe Nasso, Roberto Lorusso, Marco Moscarelli, Giuseppe De Martino, Angelo M. Dell’Aquila, Arash Motekallemi, Nicola Di Bari, Ignazio Condello, Pasquale Mastroroberto, Giuseppe Santarpino, Giuseppe Speziale

**Affiliations:** 1 Department of Cardiac Surgery, GVM Care&Research, Anthea Hospital, Via Camillo Rosalba, 35, /38 Bari, Italy; 2 Cardio-Thoracic Surgery Department, Heart & Vascular Centre, Maastricht University Medical Centre (MUMC), Maastricht, Netherlands; 3 Cardiovascular Research Institute Maastricht (CARIM), Maastricht, Netherlands; 4 Department of Cardiology and Electrophysiology, Clinica Mediterranea, Naples, Italy; 5 Department of Cardiac Surgery, Münster Universität, Münster, Germany; 6 Department of Cardiac Surgery, “Aldo Moro“ University, Bari, Italy; 7 Department of Experimental and Clinical Medicine, Magna Graecia University, Catanzaro, Italy; 8 Department of Cardiac Surgery, Paracelsus Medical University, Nuremberg, Germany

**Keywords:** Surgical ablation of atrial fibrillation, Catheter ablation of atrial fibrillation, Hybrid ablation of atrial fibrillation

## Abstract

**Background:**

The debate on the best treatment strategy for atrial fibrillation (AF) has expanded following the introduction of the so-called “hybrid procedure” that combines minimally invasive epicardial ablation with endocardial catheter ablation. However, the advantage of the hybrid approach over conventional epicardial ablation remains to be established.

**Methods:**

From June 2008 to December 2020, 609 surgical AF ablation procedures through a right minithoracotomy were performed at our institution. From 2008 to 2011, a unipolar radiofrequency (RF) device was used, whereas from 2011 to 2020 a bipolar RF device was used. In addition, between September 2016 and April 2017, 60 patients underwent endocardial completion of epicardial linear ablation. In 30 of these latter patients, surgical isolation of the Bachmann’s bundle (BB) was also performed. Starting from 2021, surviving patients at follow-up were asked to undergo electrocardiographic evaluation and left ventricular function assessment and to complete a questionnaire addressing quality of life and predisposing factors for recurrent AF.

**Results:**

The ablation procedure was completed in all patients. Upon discharge, 30 (4.9%) patients showed recurrence of AF, whereas the remaining patients (95.1%) were in sinus rhythm. All patients in whom a hybrid approach was used either with or without BB ablation were discharged in sinus rhythm. After a mean follow-up of 74 months, 122 (20%) patients developed recurrent AF, including 19.9% in whom a unipolar RF device was used, 21% in whom a bipolar RF device was used, 23% who had undergone a hybrid procedure without BB ablation and 3.3% who had undergone a hybrid procedure with BB ablation. On multivariate analysis, reduced left ventricular ejection fraction, worsening of European Heart Rhythm Association symptom class, and cognitive impairment or depression during follow-up were found to be significantly associated with AF recurrence.

**Conclusions:**

Surgical AF ablation through a right minithoracotomy is safe and may allow the creation of additional linear lesions, particularly in the BB. The placement of adjunctive linear lesions in the setting of a hybrid procedure can be more effective in reducing the risk for AF recurrence than isolated surgical ablation or hybrid ablation without the addition of further linear lesions, with no incremental risk to the patient.

## Background

Prevalence of atrial fibrillation (AF) is increasing worldwide and its management poses challenges that prompted the recent update of the European Society of Cardiology/European Association of Cardio-Thoracic Surgery guidelines [1]. Catheter ablation of AF improved significantly over the past decade, but many patients still experience recurrent AF after the procedure leading to the need for repeat ablation [2, 3]. In current guidelines, surgical ablation of AF is not recommended as first-line therapy due to the limited, though promising, available evidence [4–6], but it may be considered as initial therapy in selected patients [1]. In particular, by comparing guideline recommendations of 2016 vs 2020, catheter or surgical ablation should be considered in patients with symptomatic persistent or long-standing persistent AF (Class IIb in 2016), but only AF catheter ablation for pulmonary vein isolation is recommended for rhythm control after one failed or intolerant drug therapy (Class I in 2020) [1]. Thoracoscopic or hybrid surgical ablation for patients refractory to drug therapy or after failed percutaneous AF ablation stays as a Class IIa recommendation [1].

Recently, it has been shown that electrical isolation of the left atrial posterior wall, particularly when targeting the Bachmann’s bundle (BB), may play a key role in ensuring procedural success [7]. BB may be involved in the pathogenesis of AF by sustaining a number of unstable reentrant circuits, and BB isolation has been hypothesized to prevent induction of stable AF [7–10]. As indicated by current guidelines, the achievement of complete ablation is of great relevance. The aim of this study was to evaluate the efficacy of endoscopic ablation, with and without the creation of adjunctive linear lesions at the BB region, in preventing or detecting gaps in ablation lines in patients who have previously undergone epicardial ablation, compared to isolated surgical ablation.

## Methods

From June 2008 to December 2020, 609 consecutive patients underwent surgical or two-staged hybrid ablation at the Anthea Hospital, Gruppo Villa Maria (GVM) Care & Research, Bari, Italy, and were followed up to monitor AF recurrences. Surgical treatment of AF was performed through a right minithoracotomy using a unipolar radiofrequency (RF) device from 2008 to 2011 (Estech, Cobra Adhere XL) and a bipolar RF device (i.e. unidirectional device with two electrodes) from 2011 onward (Estech COBRA Fusion™ 150 Surgical Ablation System). All enrolled patients underwent epicardial isolation of the pulmonary veins (PVs) and the left atrial posterior wall (“box lesion”).

Our operative technique has been described elsewhere [4]. Briefly, a 3 to 4 cm right minithoracotomy was performed at the level of the 3rd intercostal space. The devices deliver bipolar or unipolar RF energy with the aim to obtain electrical PV isolation by temperature controlled RF ablation of the atrial myocardium. The ablation was performed by two energy applications lasting 150 s each. These were followed by a 60 s application after the probe was moved circumferentially, to achieve complete closure of the box lesion. All procedures were performed off-pump. A circular box lesion was created.

Among hybrid patients (*n* = 60), 30 patients underwent surgical ablation with additional linear lesions targeting the BB. The BB was ablated by introducing the magnetic tip below the ascending aorta and above the roof of the left atrium and then advanced to the base of the left atrial appendage. No further dissection was required.

As previously described [7], hybrid patients underwent a staged endocardial ablation within 6 weeks after the surgical procedure with the aim at verifying or ablating: (i) the surgical lines and completing isolation of the box lesion if needed, (ii) additional right and left atrial substrate modification, and (iii) other triggers of AF.

Catheter ablation was performed under general anesthesia and esophageal temperature monitoring with a dedicated tripolar catheter (Esotherm, Fiab). Mapping and ablation were performed using an electroanatomic mapping system (CARTO, Biosense Webster, Diamond Bar, CA, USA). A detailed bipolar voltage map of the left atrium was obtained. All points were acquired point-by-point using the ablation catheter to ensure adequate catheter tissue contact by contact force. RF was applied using an open irrigated tip catheter with power output up on the posterior wall and in the remaining atrial sites. Entrance block was defined by complete elimination or dissociation of PV potentials, determined by the circular mapping catheter positioned in the PVs and posterior wall [7]. Endocardial ablation was first directed to possible gaps in the surgical lesions. Moreover, the procedure was completed with:

- ablation of the Marshall ligament,
- roof and anterior mitral lines,
- coronary sinus and superior vena cava isolation,
- intercaval and cavotricuspid isthmus lines.

Patients were then referred to the local cardiologist with recommended follow-up at 6, 9, 12 months and then every 6 or 12 months depending on rhythm stability. Moreover, starting from 2021, surviving patients were asked to undergo assessment of left ventricular function and to complete a questionnaire addressing their behavior in relation to predisposing factors for recurrent AF, including alcohol and caffeine intake, smoking, and weight excess or loss. In addition, patients with diabetes, hypertension or obstructive sleep apnea requiring continuous positive airway pressure were asked if they were receiving appropriate treatment for their disease and were facing any difficulties in compliance and achieving optimal medical therapy. Changes in patients’ quality of life were also investigated based on New York Heart Association (NYHA) functional classification and European Heart Rhythm.

### Association (EHRA) symptom scale

In particular, the questions for the patients in the interview included the following:

- Do you have dyspnea/respiratory impairment? Can you take the stairs? How many floors?
- Are your symptoms restricting your normal daily activities? Did you have to reduce or stop your normal daily activities?
- Do you smoke or are you continuing to smoke?
- Do you drink alcohol, coffee, tea or take other stimulants?
- Have you lost or gained weight in the past few years?

The risk for recurrent AF was determined by evaluating clinical and echocardiographic parameters recorded at last follow-up on the basis of patient-reported outcomes and questionnaire assessment on postoperative behavioral modifications aimed at reducing triggering factors for AF (i.e. weight excess or loss, reduction or elimination of alcohol and caffeine intake, smoking cessation, optimal management of diabetes or hypertension or sleep apneas). Quality of life was evaluated using NYHA functional classification and the EHRA score of AF-related symptoms.

The GVM Care&Research ethics committee approved the study and all patients provided written informed consent for the procedure and study enrollment.

#### Statistical analysis

Data analysis was performed using Excel 2016 (Microsoft, Redmond, WA, USA) and statistical analysis was performed using SPSS (IBM SPSS Statistics for Windows, Version 27.0. Armonk, NY: IBM Corp). Categorical variables are given as counts and percentages. Event-free estimate such as recurrence of AF was determined using the Kaplan-Meier method. Possible risk factors for AF recurrence are reported in Table 1 and were used for determining the predictive model. To this purpose, univariate analysis was performed first. Variables with a *p*-value =0.2 were included in a multivariable model for Cox regression analysis with stepwise selection to determine the independent predictors of AF recurrence. A p-value *p* < 0.05 was considered statistically significant.

**Table 1:** Univariate and multivariate analysis

	Including Bachmann bundle		Exp (B)/ Hazard
	Univariate ***p***-value	Multivariate p-value	
Age	0.426		
Type of ablation	0.694		
Complications intraop	0.11	0.015	
Complications postop	0.056	0.929	
Rhythm	0.352		
Rhythm at discharge	0.834		
Follow-up			
Exitus	n.a.		
Date	n.a.		
Months	n.a.		
Years	n.a.		
Rhythm	n.a.		
Ejection fraction	0.453		
Ejection fraction at follow-up	0.000	0.002	0.939
NYHA class	0.000	0.637	0.848
Gained 10 kg	0.049	0.851	
Continued smoking	0.141	0.348	0.189
Continued drinking	0.000	0.155	0.970
CPAP at follow-up	0.015	0.714	0.700
Hypertension at follow-up	0.319		
Diabetes at follow-up	0.054	0.327	
Caffeine consumption	0.001	0.610	0.733
EHRA score	0.065	0.048	0.873
Hospitalization for cardiac causes	0.000	0.305	0.941
Cerebrovascular event at follow-up	0.025	0.235	1.021
Cognitive impairment or depression	0.000	0.401	0.515
**Cox Regression - Method**			
Ejection fraction at follow-up	14.099	0.000	
EHRA score	8.641	0.003	
Cognitive impairment or depression	1.275	0.259	

*CPAP* continuous positive airway pressure, *EHRA* European Heart Rhythm Association, *NYHA* New York Heart Association

## Results

Surgical ablation was completed in all patients and was performed in 324 (53.2%) patients with paroxysmal AF and 285 (46.8%) patients with persistent AF. Mean age of the study population was 63 (range 27–87) years. The AF ablation procedure was performed using a unipolar RF device in 151 (24.8%) patients and a bipolar RF device in 398 (65.2%) patients. A hybrid approach using a bipolar RF device was adopted in 60 patients with additional linear lesions targeting the BB in 30 (5%) patients.

Intraoperative and postoperative complications were recorded in 7 (1.1%) and 11 (1.8%) patients, respectively. The 30-day mortality was 0%.

At discharge, most patients (95.1%) were in sinus rhythm whereas 4.9% (*n* = 30) were in AF. No patient required pacemaker implantation. Notably, all patients who had undergone hybrid ablation either with or without BB ablation were discharged in sinus rhythm after completion of the second procedure.

**Fig. 1:**
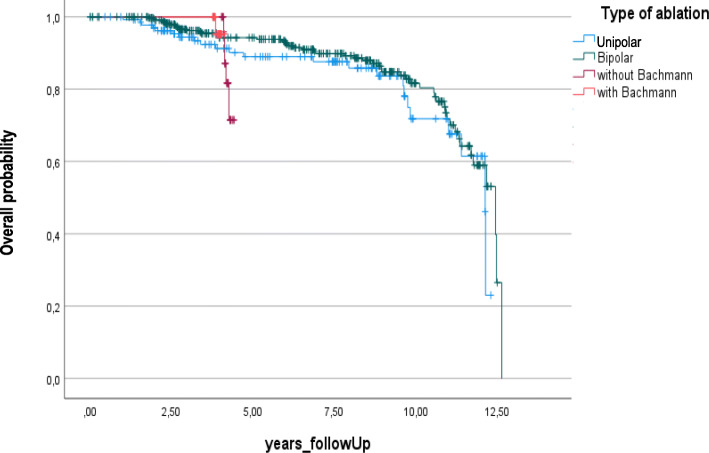
Atrial fibrillation recurrence by type of ablation procedure. Recurrence rates were 19.9% with unipolar RF ablation, 21% with bipolar RF ablation, 23% with hybrid procedure without adjunctive BB ablation, and 3.3% with hybrid procedure with adjunctive BB ablation

At a mean follow-up of 74 (2–152) months, 4 (0.6%) patients died of non-cardiac causes and 122 (20%) patients experienced recurrent AF. AF recurrence rates by type of ablation procedure are shown in Fig. 1 [unipolar RF ablation: 19.9% (*n* = 30); bipolar RF ablation: 21% (*n* = 84; hybrid procedure without adjunctive BB ablation: 23% (*n* = 7); hybrid procedure with adjunctive BB ablation: 3.3% (*n* = 1)].

On univariate analysis, intraoperative complications, persistence of AF triggers (overweight, continued alcohol and caffeine consumption), suboptimal management of continuous positive air pressure and diabetes, reduced left ventricular ejection fraction, higher NYHA class, hospitalization due to cardiac causes, the occurrence of cerebrovascular events and cognitive impairment or depression were found to be associated with recurrent AF during follow-up (Table 1). On multivariate analysis, intraoperative complications, impaired left ventricular ejection fraction, worsening of EHRA symptom class and cognitive impairment or depression during follow-up remained significantly associated with AF recurrence (Table 1).

## Discussion

Monitoring of patients undergoing surgical or endovascular ablation of AF has evolved remarkably over the last years. Current guidelines emphasize the importance of achieving complete electrical PV isolation [1], a concept that cannot be taken for granted and should not be neglected given that PV reconnection rates are as high as 70% [1].

However, despite the large number of catheter ablation procedures, only few patients undergo multidisciplinary heart team discussion for proper decision making about hybrid AF ablation. Although various ablation strategies have been proposed and implemented into clinical practice [11–15], the success rate of catheter ablation in AF patients remains low, with wide variations in ablation techniques among operators. In our study, surgical AF ablation was unsuccessful in one fifth of patients likely due to the lack of mapping and catheter ablation. Increasing evidence suggests that the hybrid approach could represent a more aggressive, but very effective treatment for such patients [16].

Given the not negligible proportion of patients experiencing failed ablations with subsequent poorer long-term clinical outcomes and quality of life, it can be speculated that hybrid surgical-catheter ablation procedures combining a minimally invasive epicardial ablation with no sternotomy and cardiopulmonary bypass with a percutaneous endocardial approach may result in improved outcomes than either procedure alone [10]. However, in our study, although all patients who had undergone two-staged hybrid ablation were discharged in sinus rhythm, we could not demonstrate the superiority of the hybrid procedure over isolated surgical ablation.

The main finding of our study was a significant reduction of AF recurrence in hybrid patients in whom adjunctive BB ablation was performed. In this respect, a few considerations are relevant: (i) BB ablation in the setting of a two-staged hybrid procedure is safe and highly effective; (ii) adding this surgical ablation target, where the BB is supposed to be anatomically located, was easy to perform without a significant increase in procedural time and without requiring further blunt dissection; and (iii) BB ablation does not increase the risk for periprocedural complications.

The results obtained were better than those recorded in other centers with hybrid procedures not targeting the BB [17–20]. This could be due to the fact that the BB may be involved in a number of unstable reentrant circuits, and we hypothesized that an effective lesion in the BB would prevent induction and maintenance of AF.

It is worth noting that current guidelines also suggest that catheter ablation should be reserved for patients with AF which remains symptomatic despite optimal medical therapy [1]. Besides the clear indication for the need of providing practitioners and institutions with tools to measure the quality of care that AF patients receive so as to identify opportunities for improvement, the impact of lesion sets in addition to PV isolation is still uncertain. The debate remains therefore open and guidelines prompt us to improve the quality of our treatment strategies. Our study contributes in that direction by performing adjunctive BB ablation with the aim to improve the outcome. Adjunctive BB ablation in the setting of a hybrid surgical approach using minithoracotomy was safe, with an intraoperative complication rate similar to hybrid surgical ablation not targeting the BB (1% vs 3%, *p* = 0.42).

Prospective, registry-based data show that approximately 4 to 14% of patients undergoing catheter AF ablation experience complications, which means that these data do not differ from those reported with thoracoscopic surgical ablation [1]. Our results show that the rate of intraoperative complications, either with or without adjunctive BB ablation, is similar to that observed with the endoscopic or thoracoscopic approach but such complications can be safely managed through a right minithoracotomy performed under direct vision. Despite being considered more invasive and burdened by higher risk, minimally invasive surgical ablation through a right minithoracotomy can also allow to address technical challenges when performing additional lesion lines (e.g. adjunctive BB ablation [9]), which seem to confer encouraging results but are not considered yet in current guidelines due to the lack of sufficient evidence*.*

In addition to safety aspects, a few considerations on efficacy deserve mentioning. The FAST trial randomized patients who were prone to AF catheter-ablation failure (i.e. failed previous ablation or left atrial dilation and hypertension) and reported common but substantially lower AF recurrence rates after thoracoscopic compared with catheter ablation (56% vs. 87%) at long-term follow-up [21]. In our study, among the 30 patients who had undergone adjunctive BB ablation, only one (3%) had recurrent AF at a mean follow-up of 47 (45–49) months.

Our study has limitations that include a small sample size and the single-center experience. However, this is the largest case series to date of patients treated by minithoracotomy and, associated in some cases with a hybrid approach with or without BB ablation. Further multicenter and randomized experiences are needed to confirm our results.

In conclusion, we believe that both isolated surgical or catheter ablation of AF are destined to provide unsatisfactory results but, at present, only few data are available for the hybrid approach. It would be interesting to understand why the high number of catheter ablation procedures is not counterbalanced by a proportionate number of hybrid procedures.

## Conclusions

The results of our study show that surgical AF ablation through a right minithoracotomy is safe and may be safer than thoracoscopic or catheter ablation according to available evidence. Higher procedural success rates could be obtained from the adoption of a hybrid approach to achieve complete closure of the box lesion with adjunctive BB ablation.

## Data Availability

The datasets used and analysed during the current study are available from the corresponding author on reasonable request.

## Abbreviations

AF: Atrial fibrillation
BB: Bachmann’s bundle
EHRA: European Heart Rhythm Association
GVM: Gruppo Villa Maria
NYHA: New York Heart Association
PV: Pulmonary vein
RF: Radiofrequency
